# Proposal of the Glaucoma Etiology Complex (GEC): A Structured Framework for Understanding the Multifactorial Nature of Glaucoma

**DOI:** 10.7759/cureus.84379

**Published:** 2025-05-19

**Authors:** Masaki Tanito

**Affiliations:** 1 Department of Ophthalmology, Shimane University Faculty of Medicine, Izumo, JPN

**Keywords:** comprehensive framework, etiology, glaucoma, patient-centered care, risk factors

## Abstract

The Glaucoma Etiology Complex (GEC) is a conceptual framework encompassing all elements involved in the pathophysiology of glaucoma. Glaucoma is no longer understood as a disease driven solely by intraocular pressure; instead, it is increasingly recognized as a complex condition influenced by ocular, systemic, genetic, vascular, and lifestyle-related factors. The nine components of the GEC are: 1. age, 2. genetic factors, 3. intraocular pressure, 4. ocular characteristics, 5. ocular comorbidities, 6. vascular factors, 7. systemic comorbidities, 8. lifestyle factors, and 9. patient treatment attitude. These components interact with each other to dynamically shape the disease process over time.

This editorial presents the structure and significance of the GEC, aiming to provide a comprehensive framework for understanding the multifactorial nature of glaucoma. This perspective may serve as a useful reference for both clinicians and researchers in pursuing individualized risk assessment, early diagnosis, and personalized management strategies for glaucoma.

## Editorial

Glaucoma has long been recognized as a leading cause of irreversible blindness worldwide. Recent high-impact publications have further expanded our understanding of its multifactorial nature and pathophysiological complexity [1-5]. Traditionally, elevated intraocular pressure (IOP) was considered the principal risk factor and therapeutic target. However, it has become increasingly clear that glaucoma is a multifactorial disease influenced by a wide array of elements extending beyond IOP alone. This shift in understanding necessitates a more comprehensive framework that can accommodate the complexity and heterogeneity of glaucoma pathogenesis.

**Figure 1:**
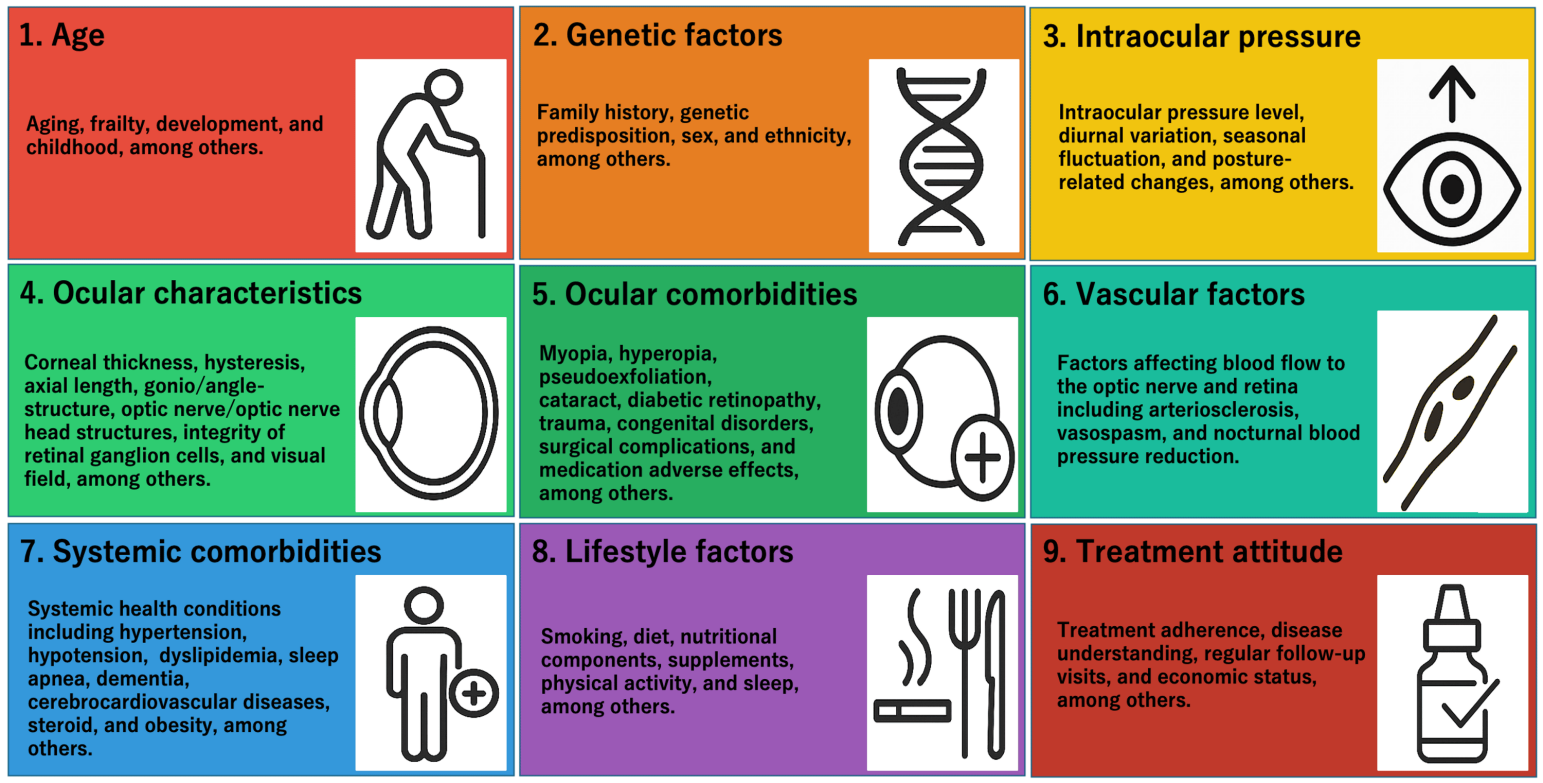
The Glaucoma Etiology Complex (GEC): nine interacting components The GEC framework illustrates the nine components of factors involved in the pathophysiology of glaucoma: 1. Age, 2. Genetic factors, 3. Intraocular pressure, 4. Ocular characteristics, 5. Ocular comorbidities, 6. Vascular factors, 7. Systemic comorbidities, 8. Lifestyle factors, and 9. Patient treatment attitude. These components interact dynamically over time, contributing to disease development, progression, and therapeutic response. Image credits: The author

To address this need, I propose the Glaucoma Etiology Complex (GEC)- a conceptual model that encompasses all factors contributing to the development and progression of glaucoma. The GEC consists of the following nine components (Figure 1).

Age

Age is one of the most significant risk factors for glaucoma. However, in the GEC framework, age-related influence is considered across the entire human lifespan. While senescence contributes to progressive damage to ocular tissues, such as the trabecular meshwork and optic nerve, early-life factors such as intrauterine or childhood developmental insults may predispose individuals to structural vulnerabilities that manifest later. Frailty, a condition of reduced systemic reserve in older adults, may further influence disease expression and treatment outcomes. Thus, age-related elements should be viewed in both developmental and degenerative contexts. Future directions include research into biological aging markers and the clinical application of comprehensive geriatric assessment within ophthalmology to assess individual vulnerability and tailor interventions.

Genetic factors

A strong family history of glaucoma is well-established as a risk factor, but emerging evidence suggests complex polygenic and epigenetic interactions underlying disease susceptibility. In addition to genetic predisposition, sex-based differences in prevalence and progression, as well as ethnic background, must be considered. Glaucoma with elevated IOP tends to be more prevalent in individuals of African descent, whereas normal-tension glaucoma is more commonly observed in Asian populations. These factors influence both the risk of disease and the response to certain treatments, highlighting the need for personalized approaches in clinical decision-making. The development of user-friendly genetic testing platforms, such as point-of-care gene panel chips, would allow for broader screening and risk stratification in clinical settings.

IOP

While elevated IOP remains the most modifiable risk factor for glaucoma, it is not the sole determinant of disease onset or progression. The GEC includes a more nuanced view of IOP-related dynamics. Diurnal fluctuations, posture-induced changes, and seasonal variations can all contribute to optic nerve stress over time. Even within the so-called "normal" range of pressure, certain individuals may experience glaucomatous damage due to heightened susceptibility, emphasizing the importance of individualized targets. The development of wearable or implantable devices that enable continuous, long-term monitoring is essential to improving disease tracking and management.

Ocular characteristics

Structural and functional attributes of the eye greatly influence glaucoma risk. These include central corneal thickness and corneal hysteresis, which affect pressure measurement accuracy and biomechanical stress transmission; axial length, which is particularly relevant in myopic eyes; and anatomical features of the angle and optic nerve head, observable via gonioscopy and imaging. Additionally, the health of retinal ganglion cells and visual field sensitivity are essential parameters for assessing disease status. The combination of these features provides a unique ocular profile for each patient. Future diagnostic innovation should focus on imaging and biomarker technologies capable of evaluating the trabecular meshwork and retinal ganglion cells at the cellular level, as well as objective, patient-independent methods for assessing visual function.

Ocular comorbidities

A variety of concurrent ocular conditions can exacerbate or mask glaucoma. Both high myopia and hyperopia alter ocular structure in ways that may complicate diagnosis and accelerate progression. Pseudoexfoliation syndrome and diabetic retinopathy involve pathological changes in the extracellular matrix and microvasculature, respectively. Cataracts may obscure optic disc evaluation and are also recognized as an important contributing factor in the development of primary angle closure disease. Previous ocular trauma or intraocular surgery can lead to secondary glaucoma. Certain ophthalmic medications, such as corticosteroids and prostaglandin analogs, may unintentionally raise IOP or cause periorbital changes, respectively. These comorbidities must be carefully integrated into individualized care plans. Pursuing treatment strategies that minimize complications and adverse effects while maximizing therapeutic efficacy is a critical direction for future research. It is essential to pursue treatment approaches that minimize complications and adverse effects while maximizing therapeutic efficacy.

Vascular factors

Adequate perfusion of the optic nerve head and retina is essential for neuronal survival. Vascular dysregulation, including nocturnal hypotension, arteriosclerosis, and vasospasm, can compromise blood flow, increasing the risk of glaucomatous damage. In some patients, vascular factors may play a more dominant role than IOP itself. Clinicians should be aware of systemic vascular conditions and monitor blood pressure patterns, especially in those with normal-tension glaucoma or progressive disease despite IOP control. Although vascular imaging has advanced, standardizing ocular perfusion evaluation remains a challenge and a future research target.

Systemic comorbidities

A wide range of systemic diseases is associated with glaucoma risk and progression. Hypertension, hypotension, dyslipidemia, sleep apnea, and cerebrovascular diseases have all been implicated through shared mechanisms such as endothelial dysfunction and oxidative stress. Neurological conditions like dementia may affect patient compliance and visual field interpretation. The use of systemic corticosteroids and the presence of obesity further complicate management. Understanding these comorbidities helps guide both ocular and systemic interventions. Introducing 24-hour ambulatory blood pressure monitoring into ophthalmic practice is a critical step. Moreover, systemic assessment tools tailored to ophthalmology are needed to fully capture the interplay between systemic health and glaucoma.

Lifestyle factors

Emerging evidence points to modifiable lifestyle behaviors as contributors to glaucoma risk. Smoking, poor diet, physical inactivity, and inadequate sleep are all associated with systemic inflammation and vascular dysfunction, which, in turn, affect ocular health. Nutritional components and supplements may also influence oxidative stress or neuroprotection. Lifestyle modification should be incorporated into patient counseling, especially in early-stage or high-risk individuals. A standardized, evidence-based lifestyle intervention model tailored specifically for glaucoma patients should be established to support preventive strategies and enhance patient outcomes.

Patient treatment attitude

Ultimately, the success of glaucoma management hinges on patient behavior. Adherence to prescribed medications, comprehension of disease severity, regular attendance at follow-up visits, and the ability to afford care are critical to preventing vision loss. Socioeconomic barriers, health literacy, and psychological factors, such as denial or depression, may all influence patient engagement. The GEC emphasizes that supporting the patient beyond clinical parameters is essential for long-term success.

These nine components are not isolated; they interact dynamically over time, shaping individual trajectories of disease expression and treatment response. The GEC offers a comprehensive, integrative framework that reflects the true complexity of glaucoma. It encourages clinicians to evaluate each patient holistically and promotes research that explores the multifactorial nature of the disease.

Advantages of GEC

The possible advantages of GEC include: 1. It highlights that glaucoma arises from interactions among diverse systemic and ocular factors, rather than a single cause; 2. It allows for individualized assessment of contributing elements, supporting risk stratification and personalized treatment strategies; It offers a structured framework for research, enabling a comprehensive analysis of pathophysiological mechanisms and epidemiological associations; It provides educational value in helping clinicians and trainees understand the complex nature of glaucoma

Limitations of GEC

While GEC may offer a useful conceptual framework, several limitations must be acknowledged. The definitions and relative contributions of individual components remain insufficiently established, limiting their immediate clinical applicability. Moreover, due to overlaps among some factors and the lack of standardized methods for quantifying certain components, future development of objective assessment tools and simplified evaluation systems is essential.

Conclusion

The purpose of introducing GEC is not to replace current diagnostic criteria or treatment guidelines but to supplement them with a more nuanced understanding of disease etiology. By recognizing glaucoma as a systemic, lifelong condition influenced by diverse and interacting factors, the GEC provides a foundation for more precise, personalized care. In doing so, it emphasizes the need to look beyond IOP and address the full spectrum of contributors to this complex disease.

**Figure 2:**
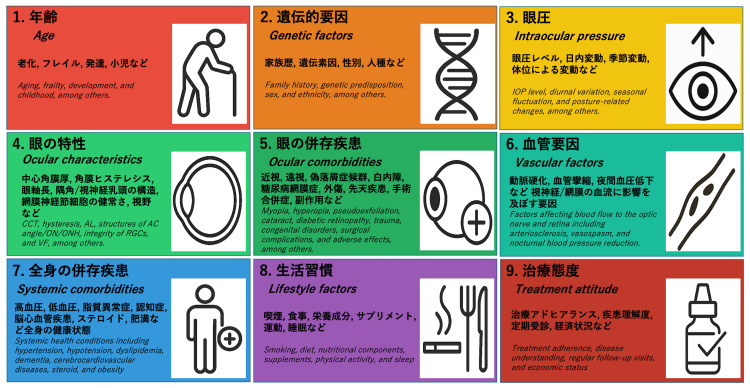
Glaucoma Etiology Complex (GEC): Japanese-English Bilingual Version

The concept of GEC was originally presented in Japanese during the 129th Annual Meeting of the Japanese Ophthalmological Society held on April 19, 2025, in Tokyo. Therefore, the original figure is provided here to illustrate the concept (Figure 2).
